# Supplementary material for: Estimating the impact of COVID-19 vaccine inequities: a modeling study
Source: Nat Commun. 2023 Jun 6;14:3272. doi: 10.1038/s41467-023-39098-w (PMC10241610; doi:10.1038/s41467-023-39098-w)
Supplement: Supplementary file 1 — Supplementary Information [file 41467_2023_39098_MOESM1_ESM.pdf]

Supplementary Material for *Estimating the impact of COVID-19 vaccine inequities: a modeling study*

Nicolò Gozzi[1,2], Matteo Chinazzi[3], Natalie E. Dean[4], Ira M. Longini Jr.[5], M. Elizabeth Halloran[6,7], Nicola Perra[8,3], Alessandro Vespignani[3]

[1] Networks and Urban Systems Centre, University of Greenwich, UK
[2] ISI Foundation, Turin, Italy
[3] Laboratory for the Modeling of Biological and Socio-technical Systems, Northeastern University, Boston, MA USA
[4] Department of Biostatistics and Bioinformatics, Emory University, Atlanta, USA
[5] Department of Biostatistics, College of Public Health and Health Professions, University of Florida, Gainesville, FL, USA
[6] Fred Hutchinson Cancer Center, Seattle, WA, USA
[7] Department of Biostatistics, University of Washington, Seattle, WA, USA
[8]School of Mathematical Sciences, Queen Mary University, London, UK

May 19, 2023

Contents

1 Data **2**

2 Epidemic model **2**

3 Model calibration **4**

4 Non-pharmaceutical interventions **8**

5 Modeling the introduction of a second SARS-CoV-2 strain **9**

6 Counterfactual scenarios **9**
6.1 Vaccination rates of high income settings . . . 9
6.2 Earlier start of factual vaccinations . . . 11
6.3 NPIs increase . . . 11

7 Estimating the impact of the factual vaccination campaigns **12**

8 Model with time-varying reporting of deaths **15**

9 Countries demographic **18**

1 Data

Demographic and epidemic data. Data about demographics comes from the United Nation World Population Prospects [1]. Epidemiological data are taken from the COVID-19 Data Repository by the Center for Systems Science and Engineering (CSSE) at Johns Hopkins University and from official sources [2].

Vaccination data. Data on global vaccine inequities used in the Introduction and in Fig. 1 of the main text are taken from the United Nations Development Programme via their Global Futures Platform [3]. Vaccination data used in the simulations are taken from Our World in Data [4]. The dataset provides the cumulative share of people partially (p_t) and fully (f_t) vaccinated against SARS-CoV-2 at time t. We turn these two quantities into daily number of administered doses. Consider the cumulative fraction of partially vaccinated individuals p_t. First, we fix possible null values using a linear interpolation. Second, we turn p_t into the actual number of partially vaccinated P_t by simply multiplying it by the total population N of the country (i.e., $P_t = N \times p_t$). Finally, we take daily increases in the cumulative number of partially vaccinated individuals to get the number of daily first doses administered in the country. Through analogous calculations we get the number of daily second doses administered. In Fig. 1-A we show the percentage of individuals partially and fully vaccinated in the countries considered.

2 Epidemic model

We adopt a SEIR-like compartmental model (see Figure 2 for a schematic depiction). The susceptible individuals are placed in the compartment S. Getting in contact with the Infectious (I) they transition to the compartment of the Latent (L). Latent individuals are infected but become infectious only after ϵ^{-1} days when they eventually pass to the compartment I. After μ^{-1} days, infectious subjects finally transition to the compartment of the Recovered (R). By considering the COVID-19 characteristics we set $\epsilon^{-1} = 4days$ and $\mu^{-1} = 2.5days$. [5, 6]. We compute the number of deaths on daily recovered. In particular, the individuals that exit from the I compartment, can either transition to the Recovered compartment (R) or the Dead compartment (D). The share of individuals transitioning to the D compartment is regulated by the age-stratified Infection Fatality Rate (IFR) from Ref. [7]. To account for possible delays due to hospitalization and reporting between the transition $I \to R$ and actual death, we record the number of deaths computed on the recovered of day t only after Δ days. In other words, D individuals transition to the compartment D^o (superscript o stands for "observed") at a rate $1/\Delta$. Individuals are divided into 10 age groups ($0-9$, $10-19$, $20-24$, $25-29$, $30-39$, $40-49$, $50-59$, $60-69$, $70-79$, $80+$). The age-stratified rate of interaction are defined by the country specific contacts matrix C from Ref. [8].

We also introduce a seasonal term to capture modulation of the force of infection regulated by changes in factors such as temperature and humidity [9, 10]. This means that in our simulation R_t is multiplied by a rescaling factor $s_i(t)$ defined as $s_i(t) = \frac{1}{2}\left[\left(1 - \frac{\alpha_{min}}{\alpha_{max}}\right)\sin\left(\frac{2\pi}{365}(t - t_{max,i}) + \frac{\pi}{2}\right) + 1 + \frac{\alpha_{min}}{\alpha_{max}}\right]$, where i refers to the hemisphere considered, and $t_{max,i}$ is the day associated to the maximum of the rescaling function. For the northern hemisphere it is set to January 15th and to July 15th for the southern hemisphere, while we consider no seasonal modulation in the tropical hemisphere. If a country extends across multiple zones, the seasonal factor is a weighted average of the different $s_i(t)$ according to the population living in the different hemispheres. We fix $\alpha_{max} = 1$ and consider α_{min} as a free parameter (more details are provided in Section 3).

In these settings, we model both vaccinations and the introduction of a second, more transmissible virus strain. In particular, individuals who received one dose of vaccine move to the compartments denoted with the superscript V_1. We assume that all individuals except for the infectious can receive the vaccine. Hence, susceptible, latent and recovered are vaccinated proportionally to their number. For S^{V_1} individuals the force of infection is reduced by a factor $(1 - VE_{S1})$. If these individuals get infected, their IFR is also reduced by a factor $1 - VE_{M1}$. It follows that, in our simulations, the overall efficacy of a single dose of vaccine against death is $VE_1 = 1 - (1 - VE_{S1})(1 - VE_{M1})$. After receiving the second dose, individuals transition to the compartments with superscript V_2. Similarly, force of infection and IFR for them is reduced, respectively, by $(1 - VE_{S2})$ and $(1 - VE_{M2})$, implying an overall efficacy of $VE_2 = 1 - (1 - VE_{S2})(1 - VE_{M2})$. We also assume that vaccinated individuals that get infected are less infectious by a factor $(1 - VE_I)$ [11]. Since vaccine protection is not immediate, we introduce a delay of Δ_V days between administration (of both 1st and 2nd dose) and actual effect of the vaccine.

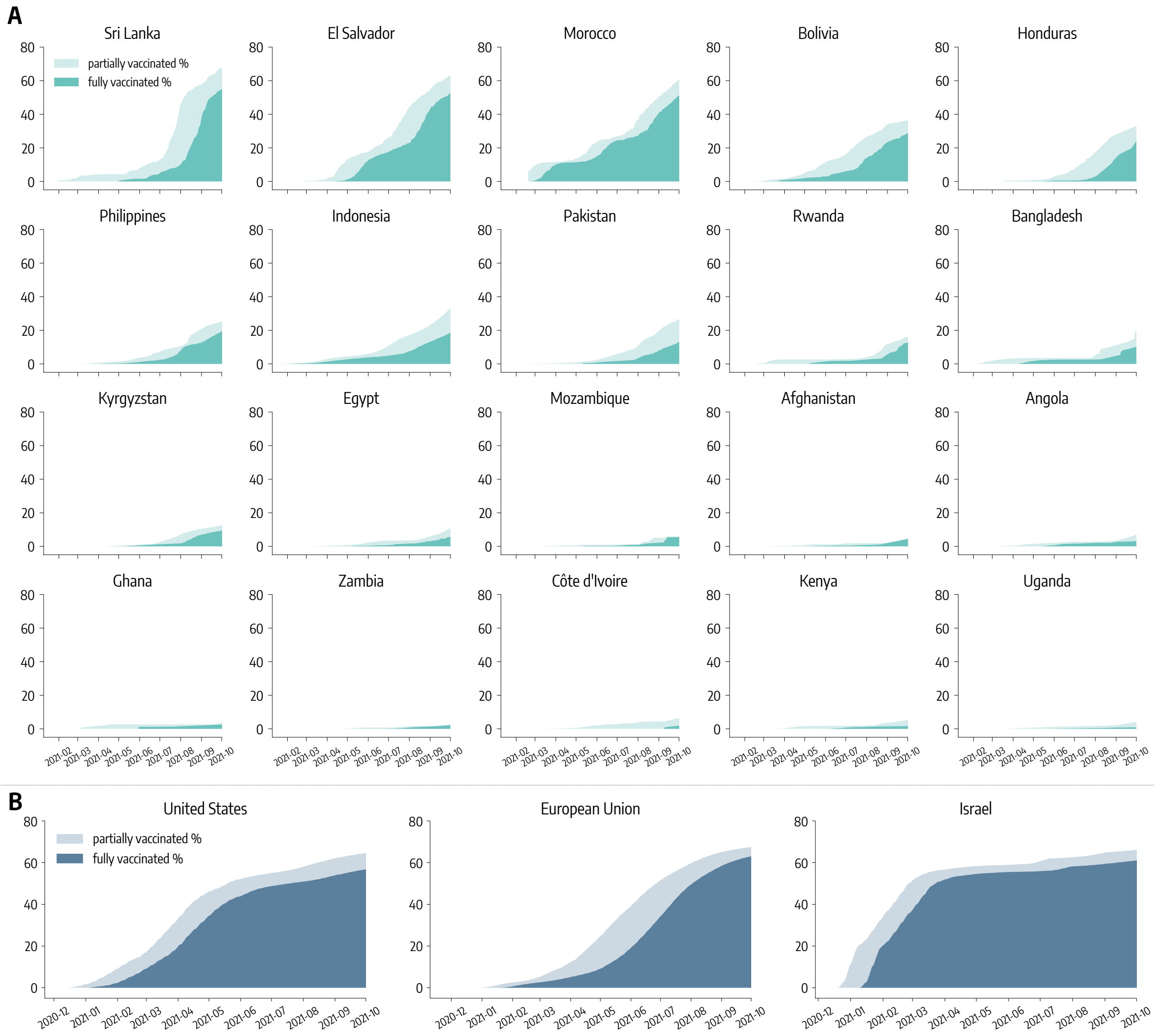

Supplementary Figure 1: **Vaccinations.** A) Percentage of partially and fully vaccinated individuals in LMIC up to 2021/10/01. B) Percentage of partially and fully vaccinated individuals in high income settings up to 2021/10/01.

For example, an individual who received the 1st dose on day t, will be protected with efficacy VE_1 only, on average, after Δ_V days. Hence, the transitions to compartments with superscript V_1 and V_2 take place at rate Δ_V^{-1} after first and second inoculation. We set $\Delta_V = 14days$. We do not have detailed information about the age of individuals receiving vaccines in all the countries considered. Therefore, we assume that the rollout proceeds prioritizing the elderly. We note how this is the strategy followed by the vast majority of governments worldwide [12–14]. This means that, in our model, vaccines are distributed in decreasing age order until all 50+ individuals are vaccinated, after vaccines are distributed homogeneously to the age groups $10 - 50$. We inform the model with the number of daily 1st and 2nd doses in different countries from Ref. [15]. In this work we set $VE_1 = 80\%$ ($VE_{S1} = 70\%$), $VE_2 = 90\%$ ($VE_{S2} = 80\%$), and $VE_I = 40\%$ [11].

We add specific L and I compartments to account for the introduction and emergence of a variant of concern. Considering the period under examination and the evidence from genomic surveillance in all countries under examination we consider the arrival and spread of the SARS-CoV-2 variant of concern Delta. Looking at genomic sequence data from Ref. [16–18] we get a proxy date for its introduction (more details provided below). We imagine that Delta is ψ times more transmissible than the strain circulating previously and has a shorter latent period $\epsilon_{Delta}^{-1} = 3days$ [19]. We also assume that vaccines have a reduced efficacy against Delta VOC: $VE_1^{Delta} = 70\%$ ($VE_{S1}^{Delta} = 30\%$), $VE_2^{Delta} = 90\%$ ($VE_{S2}^{Delta} = 60\%$) [11]. Notice how, to avoid complicating further the model, we decided not to use a separate IFR for the Delta variant, even tough some studies pointed out that it might be more severe than other previously circulating strains [20]. This limitation is partly solved by the calibration step in which we fit a country-specific IFR multiplier (more details are provided in Section 3).

The model is stochastic and transitions among compartments are simulated through chain binomial processes. More in detail, at time step t the number of individuals in age group k and compartment X transiting to compartment Y is sampled from $Pr^{Bin}(X_k(t), p_{X_k \to Y_k}(t))$, where $p_{X_k \to Y_k}(t)$ is the transition probability. As an illustrative example consider the number of new infected individuals from the S compartment. The rate for this transition is called *force of infection* (generally referred as λ) and may depend on several factors, from transmissibility of the pathogen to contact rates and seasonality. In our case, the force of infection for age group k at time t is defined as:

$$\lambda_k(t) = \beta \times s(t) \times r(t) \times \left\{ \sum_{k'} \frac{C_{kk'}}{N_{k'}} \left[I_{k'} + (1 - VE_I)(I_{k'}^{V1} + I_{k'}^{V2}) \right] \right\} \tag{1}$$

Where β is the transmission rate, $s(t)$ is the seasonality factor, $r(t)$ captures contacts reduction due to NPIs (more details below), and the term in brackets is the probability of contacting an infectious individual in age group k' given the contact rates and the number of individuals per group. Notice how infectious individuals that received a vaccine (either one or two dose) have their infectiousness reduced by the $(1 - VE_I)$ factor. The probability of the infection transition is simply the force of infection multiplied by the length of the simulation step Δt (i.e., $p_{S_k \to L_k}(t) = \lambda_k(t)\Delta t$). Here, the unit of time of the simulation is the day, therefore $\Delta t = 1$. The number of S_k individuals getting infected at time t is then extracted from $Pr^{Bin}(S_k(t), \lambda_k(t))$. Given this, we can easily get the infection probability also for susceptible individuals that received one or two vaccine doses, namely S^{V_1} and S^{V_2}. Indeed, since vaccines offer a protection VE_S from infection, we have that $p_{S_k^{V_1} \to L_k^{V1}}(t) = (1 - VES_1) \times \lambda_k(t)$ and $p_{S_k^{V_2} \to L_k^{V2}}(t) = (1 - VES_2) \times \lambda_k(t)$.

3 Model calibration

The free parameters of the model are calibrated through an Approximate Bayesian Computation based on Sequential Monte Carlo [21, 22]. The ABC-SMC is an extension of the more simple rejection algorithm, which works as follow. A prior distribution $P(\theta)$ is defined for the free parameters θ. At each step of the iterative algorithm, a set of parameters $\hat{\theta}$ is sampled from $P(\theta)$ and an instance of the model is generated using $\hat{\theta}$. Then, an output quantity of the model E' is compared to the corresponding real quantity E using an error metric $S(E, E')$: if $S(E, E')$ is smaller than a tolerance δ, $\hat{\theta}$ is accepted otherwise is rejected. This process is repeated iteratively until P parameter sets are accepted. The main limit of this approach is that the acceptance criterion is never updated, leading to slow convergence. Additionally, it is often difficult to find, a priori, a reasonable value for the tolerance δ. This is particularly problematic in a case like ours, where several models need to be calibrated. The ABC-SMC algorithm approach used in this work solves these issues by implementing a sequence of rejection steps (generations) that are

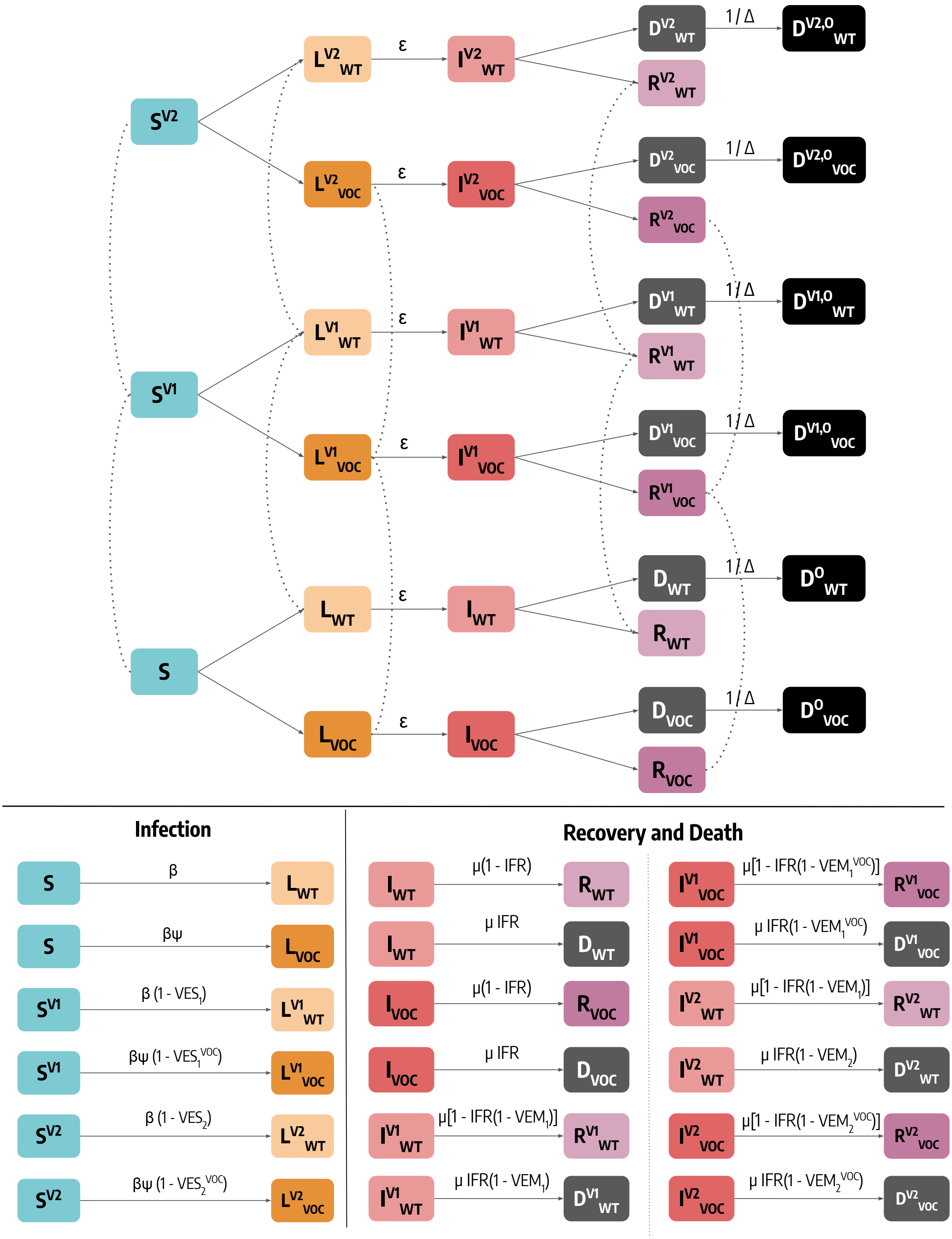

Supplementary Figure 2: **Schematic representation of the epidemic model.** For simplicity, we represent the model for a single age group. Dashed lines indicate data-driven transitions linked to vaccination status, solid lines indicate that simulated transitions. In the bottom of the figure we report the rate of transitions related to both infection and and recovery/death.

increasingly less tolerant and precise. Indeed, the prior distribution of a step is the posterior distribution (i.e., the accepted parameters) of the previous one perturbed through a kernel function to avoid local minima. This allows us to start from high error tolerances and wide prior distributions and explore more and more accurately the interesting regions of the parameter phase space as we proceed.

In this work we consider weekly deaths as output quantity and the weighted mean absolute percentage error (wMAPE) as distance metric. For each model, we consider 20 ABC-SMC generations, each with a population size of 1000 accepted parameter sets. We also set the maximum training time for each model to 24 hours. As already mentioned in the Methods section, the free parameters and the initial priors are:

- the transmission rate β; we explore uniformly values such that the R_t on the first simulation date is between 0.6 and 2.0;
- the delay in deaths $\Delta \sim U(10, 35)$ [23];
- the seasonality parameter $\alpha_{min} \sim U(0.5, 1.0)$ (0.5 indicates strong seasonality while 1.0 absence of seasonality);
- the initial number of infected individuals; we explore uniformly values between 1 and 1000 times the number of cases notified in the 7 days prior the beginning of the simulation (Inf_{start}^{mult}). We divide these individuals in the infected compartments (L, I) proportionally to the time spent there by individuals (ϵ^{-1} for L and μ^{-1} for I);
- the initial number of recovered; we explore uniformly values between 1 and 100 times the total number of reported cases up to the start of the simulation (Rec_{start}^{mult});
- the relative transmissibility advantage of the Delta VOC $\psi \sim U(1.0, 3.0)$;
- the date of the introduction of the Delta VOC. We consider values between 45 days before and after the date when Delta was responsible for at least 5% of sequenced samples according to the data from Ref. [16], more details below;
- the IFR multiplier $\sim U(0.5, 2.0)$; this number multiplies the IFR from Ref. [7];
- the percentage of deaths reported $\sim U(1\%, 100\%)$;

The model is calibrated separately for different countries during the period 2020/10/01 – 2021/10/01. The ABC-SMC calibration is implemented using the Python library *pyabc* [24]. In Tab. 1, 2, 3, 4, 5 we report the posterior distributions (median and interquartile range) of the free parameters. We also report the wMAPE of the posterior samples (i.e., our measure of goodness of fit) computed with respect to the actual number of weekly deaths.

	**Sri Lanka**	**El Salvador**	**Morocco**	**Bolivia**
R_t^{start}	1.75 [1.74, 1.77]	1.1 [0.99, 1.23]	1.16 [1.08, 1.26]	1.03 [1.0, 1.08]
Δ	27 [26, 29]	16 [13, 20]	15 [12, 18]	21 [19, 23]
α_{min}	0.73 [0.62, 0.85]	0.77 [0.65, 0.88]	0.75 [0.68, 0.83]	0.74 [0.64, 0.85]
ψ	2.72 [2.63, 2.81]	1.56 [1.46, 1.65]	2.14 [2.0, 2.29]	1.67 [1.33, 2.16]
Initial inf.per 10k	12.8 [9.0, 17.4]	126.7 [108.5, 146.0]	231.3 [197.3, 271.7]	11.3 [10.2, 12.6]
Initial rec. per 10k	72 [40, 110]	2268 [1430, 3080]	1855 [1272, 2421]	938 [628, 1330]
IFR Multiplier	0.94 [0.74, 1.23]	1.16 [0.9, 1.44]	1.03 [0.81, 1.3]	1.79 [1.69, 1.88]
% deaths reported	30.82 [23.88, 38.89]	48.5 [38.27, 60.52]	36.75 [29.14, 45.21]	89.08 [83.78, 93.58]
Date intro. VOC	04-23 [04-21, 04-24]	06-16 [06-05, 06-23]	04-24 [04-17, 05-02]	08-31 [08-19, 09-13]
wMAPE	0.36	0.20	0.21	0.17

Supplementary Table 1: **Posterior distributions of free parameters obtained via ABC calibration (Sri Lanka, El Salvador, Morocco, Bolivia)**. We show median and interquartile range of the different parameters. Dates are represented with a $mm - dd$ format and refer all to the year 2021.

	**Honduras**	**Philippines**	**Indonesia**	**Pakistan**
R_t^{start}	1.22 [1.09, 1.39]	1.02 [0.96, 1.1]	1.14 [1.12, 1.17]	0.92 [0.89, 0.95]
Δ	25 [19, 29]	25 [20, 30]	27 [22, 31]	25 [23, 28]
α_{min}	0.77 [0.63, 0.88]	0.74 [0.62, 0.85]	0.76 [0.62, 0.88]	0.91 [0.89, 0.93]
ψ	1.81 [1.72, 1.91]	1.26 [1.15, 1.39]	1.53 [1.42, 1.7]	1.1 [1.06, 1.16]
Initial inf.per 10k	53.4 [42.8, 65.8]	17.9 [13.3, 23.4]	7.8 [5.6, 10.2]	45.2 [39.3, 52.0]
Initial rec. per 10k	2517 [1655, 3441]	1247 [722, 1860]	578 [364, 768]	658 [405, 948]
IFR Multiplier	1.21 [0.98, 1.46]	1.58 [1.32, 1.77]	1.34 [1.08, 1.64]	1.12 [0.82, 1.55]
% deaths reported	61.81 [51.84, 75.37]	35.97 [30.76, 40.25]	65.11 [52.03, 80.15]	47.63 [35.45, 62.36]
Date intro. VOC	06-16 [06-09, 06-22]	06-26 [06-05, 07-10]	05-07 [04-21, 05-18]	05-27 [05-03, 06-19]
wMAPE	0.25	0.41	0.12	0.14

Supplementary Table 2: **Posterior distributions of free parameters obtained via ABC calibration (Honduras, Philippines, Indonesia, Pakistan)**. We show median and interquartile range of the different parameters. Dates are represented with a $mm - dd$ format and refer all to the year 2021.

	**Rwanda**	**Bangladesh**	**Kyrgyzstan**	**Egypt**
R_t^{start}	1.18 [1.17, 1.2]	0.85 [0.82, 0.9]	1.28 [1.13, 1.48]	0.94 [0.92, 0.96]
Δ	13 [12, 16]	19 [15, 24]	23 [17, 30]	20 [16, 25]
α_{min}	0.75 [0.66, 0.85]	0.75 [0.67, 0.85]	0.65 [0.59, 0.73]	0.62 [0.6, 0.64]
ψ	1.15 [1.07, 1.29]	1.75 [1.57, 2.06]	1.62 [1.5, 1.75]	1.17 [1.1, 1.26]
Initial inf.per 10k	0.4 [0.3, 0.5]	90.4 [56.2, 127.1]	41.7 [20.6, 68.1]	8.4 [7.4, 9.5]
Initial rec. per 10k	177 [115, 251]	1246 [837, 1586]	2162 [1277, 3283]	467 [252, 689]
IFR Multiplier	1.29 [1.04, 1.57]	1.01 [0.79, 1.37]	1.28 [0.94, 1.63]	1.69 [1.45, 1.83]
% deaths reported	44.39 [31.45, 61.43]	14.6 [11.01, 18.91]	48.72 [32.85, 66.88]	33.53 [29.69, 36.77]
Date intro. VOC	04-20 [04-06, 05-30]	04-04 [03-13, 04-26]	02-24 [02-16, 03-13]	05-27 [05-06, 06-13]
wMAPE	0.37	0.34	0.31	0.23

Supplementary Table 3: **Posterior distributions of free parameters obtained via ABC calibration (Rwanda, Bangladesh, Kyrgyzstan, Egypt)**. We show median and interquartile range of the different parameters. Dates are represented with a $mm - dd$ format and refer all to the year 2021.

	**Mozambique**	**Afghanistan**	**Angola**	**Ghana**
R_t^{start}	1.35 [1.32, 1.39]	0.93 [0.89, 0.98]	1.11 [1.09, 1.12]	1.41 [1.36, 1.45]
Δ	29 [26, 32]	11 [10, 12]	29 [23, 32]	29 [25, 32]
α_{min}	0.71 [0.61, 0.82]	0.64 [0.58, 0.72]	0.75 [0.64, 0.88]	0.73 [0.63, 0.84]
ψ	1.26 [1.17, 1.36]	1.81 [1.73, 1.89]	2.48 [2.35, 2.64]	1.62 [1.54, 1.7]
Initial inf.per 10k	1.9 [1.5, 2.4]	16.6 [9.4, 23.6]	5.2 [4.4, 5.8]	3.8 [2.5, 5.4]
Initial rec. per 10k	152 [82, 208]	624 [374, 795]	18 [10, 28]	804 [428, 1079]
IFR Multiplier	1.05 [0.79, 1.36]	1.04 [0.8, 1.41]	1.01 [0.78, 1.36]	0.99 [0.77, 1.27]
% deaths reported	5.7 [4.54, 7.59]	23.39 [17.73, 29.91]	3.49 [2.71, 4.56]	2.18 [1.71, 2.78]
Date intro. VOC	04-07 [03-19, 05-05]	03-22 [03-19, 03-26]	06-30 [06-27, 07-03]	04-29 [04-21, 05-09]
wMAPE	0.18	0.29	0.37	0.32

Supplementary Table 4: **Posterior distributions of free parameters obtained via ABC calibration (Mozambique, Afghanistan, Angola, Ghana)**. We show median and interquartile range of the different parameters. Dates are represented with a $mm - dd$ format and refer all to the year 2021.

	**Zambia**	**Côte d'Ivoire**	**Kenya**	**Uganda**
R_t^{start}	1.25 [1.21, 1.29]	1.43 [1.41, 1.45]	1.16 [1.14, 1.18]	0.9 [0.86, 0.94]
Δ	25 [20, 30]	29 [23, 34]	28 [25, 29]	25 [21, 29]
α_{min}	0.8 [0.7, 0.89]	0.74 [0.62, 0.86]	0.76 [0.63, 0.87]	0.76 [0.65, 0.87]
ψ	1.43 [1.31, 1.56]	2.28 [2.11, 2.48]	1.41 [1.37, 1.46]	1.72 [1.61, 1.81]
Initial inf.per 10k	11.1 [8.4, 15.1]	2.4 [1.9, 2.8]	5.4 [4.6, 6.3]	26.4 [14.5, 42.4]
Initial rec. per 10k	335 [201, 521]	115 [64, 163]	391 [221, 532]	98 [55, 136]
IFR Multiplier	1.19 [0.91, 1.49]	0.81 [0.67, 0.95]	0.97 [0.74, 1.29]	0.71 [0.61, 0.85]
% deaths reported	17.58 [13.72, 22.07]	1.67 [1.41, 1.99]	7.28 [5.44, 9.79]	28.21 [24.08, 35.19]
Date intro. VOC	03-15 [03-04, 04-04]	06-22 [06-15, 06-30]	05-14 [05-06, 05-21]	02-06 [02-01, 02-14]
wMAPE	0.26	0.41	0.29	0.50

Supplementary Table 5: **Posterior distributions of free parameters obtained via ABC calibration (Zambia, Côte d'Ivoire, Kenya, Uganda)**. We show median and interquartile range of the different parameters. Dates are represented with a $mm - dd$ format and refer all to the year 2021.

4 Non-pharmaceutical interventions

We model the effects of non-pharmaceutical interventions (NPIs) on contacts using the COVID-19 Community Mobility Report By Google [25]. The dataset provides, for various countries and spatial resolutions, a percentage change in individuals visiting specific locations. Here, we compute an overall mobility reduction $r(t)$ by taking the average of the fields about workplaces, retail and recreation, and transit stations. We convert this quantity into a contacts reduction parameters $c(t)$ following the relation: $c(t) = (1 + r(t)/100)^2$. Indeed, under an homogeneous assumption the number of contacts scale with the square of the number of individuals. For example, a percentage reduction of -20% translates into a contacts reduction factor of 0.64. In the simulations the contacts matrix C is multiplied by this reduction parameter $c(t)$ to account for the modulation in contacts induced by NPIs. Mobility data have been widely and successfully used during the COVID-19 Pandemic to account for the modulating effect of behaviour change on disease evolution in epidemic models [26–30]. The great success of such datasets

is partly due to the wide availability across geographies and time, thus offering detailed and standardised data that can be applied to different contexts. Nonetheless, we also acknowledge the limitation of mobility data that, due to its nature, disregards the impact of NPIs not directly impacting movements, such as face masks. Here we do not include explicitly the effect of face masks and similar NPIs, but we calibrate the initial R_t. Therefore, although not in a time-varying manner, the attenuating effect of these NPIs is factored into the transmission parameter.

5 Modeling the introduction of a second SARS-CoV-2 strain

We model the introduction of a second SARS-CoV-2 strain considering genomic sequencing data from CoVariants [16]. The data provides the fraction of processed samples by virus variant in different countries. As clear from the plots, in the period and countries under examination we observed the introduction and rapid growth of the Delta variant of concern. To reduce the impact of noise on seeding date estimates, for each country, we fit a logistic curve of the type $\hat{s}(t) = 1/(1 + e^{-\gamma(t-t_{1/2})})$ to the real fractions of Delta variant samples $s(t)$. The fit is performed via least square using the python library *scipy* [31]. In Fig. 3 we show the actual Delta prevalence (i.e., fraction of samples that fall into the Delta group) to the fitted prevalence. We also show the total number of samples processed in each week. In the case of countries for which genomic data is not available, we perform the logistic fit on all the samples from neighbouring countries for which data is available.

After the fit, we pick the first date on which the fitted Delta prevalence is greater or equal to 5% (i.e., $t \mid \hat{s}(t) \geq 0.05$). When running the simulations, on that date we calculate the 5% of the daily simulated infected individuals and we use them to initialize the compartment of the infected with the Delta variant. We choose a 5% threshold to avoid a prevalence that is too low and therefore more affected by noise. At the same time, we did not choose a higher prevalence threshold to avoid imposing a strong discontinuity on the R_t on the simulations.

6 Counterfactual scenarios

6.1 Vaccination rates of high income settings

We propose scenarios in which the LMIC considered manage the same vaccines availability of three high income contexts: United States (shown in the main text), European Union, and Israel (shown here). To do so, we run simulations in which, instead of the factual vaccination data of the LMIC considered, we use the daily number of first and second doses administered in the three high income settings. To account for different population sizes among geographical regions, we rescale the number of doses available in the counterfactual. For example, consider the case when we apply to Mozambique the vaccination rates of Israel. If Israel administered X_t^{Israel} doses on day t, in the counterfactual scenario we administer $X_t^{\prime Mozambique} = X_t^{Israel} \frac{N_{Mozambique}}{N_{Israel}}$. In Fig. 1-B we show the percentage of partially and fully vaccinated in the three high income settings. Vaccinations started on 2020/12/14 in US, on 2020/12/27 in most of European countries, and on 2020/12/19 in Israel. As of 2021/10/01, the European Union shows the highest percentage of fully vaccinated (63%), followed by Israel (61%), and US (57%). Nonetheless, we acknowledge differences among the three vaccination rollout especially at the beginning. Indeed, we see that in Israel and, to a lower extent also in the US, vaccinations were much faster after the start respect to the European Union. For example, the percentage of fully vaccinated on the 2021/03/01 was: 2.6%, 9.2%, and 37.3% in, respectively, EU, US, and Israel.

In Fig. 4, we show the percentage of deaths that are averted applying to LMIC the vaccination rates of US, EU, and Israel with respect to simulations with factual doses. The overall picture presented in the main text for US vaccination rates holds also with EU and Israel rates. Indeed, in both cases we observe that additional doses of vaccine bring a huge benefit in terms of reduction of fatalities. When considering dose availability of Israel, averted deaths in LMIC span between 60% to nearly 100%, while these figures lie between 40% and 90% when EU rates are considered. We also note that the ordering of the countries is almost the same when considering the three different rates. Across the LMIC considered, the greatest decrease in deaths is achieved by Israel rates, while EU rates are the least effective at reducing the number of deaths. A possible explanation is that, as noted previously, while EU reached a higher vaccine coverage as of 2021/10/01, the vaccine rollout in Israel was much faster in the early months of 2021, allowing to provide significant level of protection at the population level quicker. The results obtained

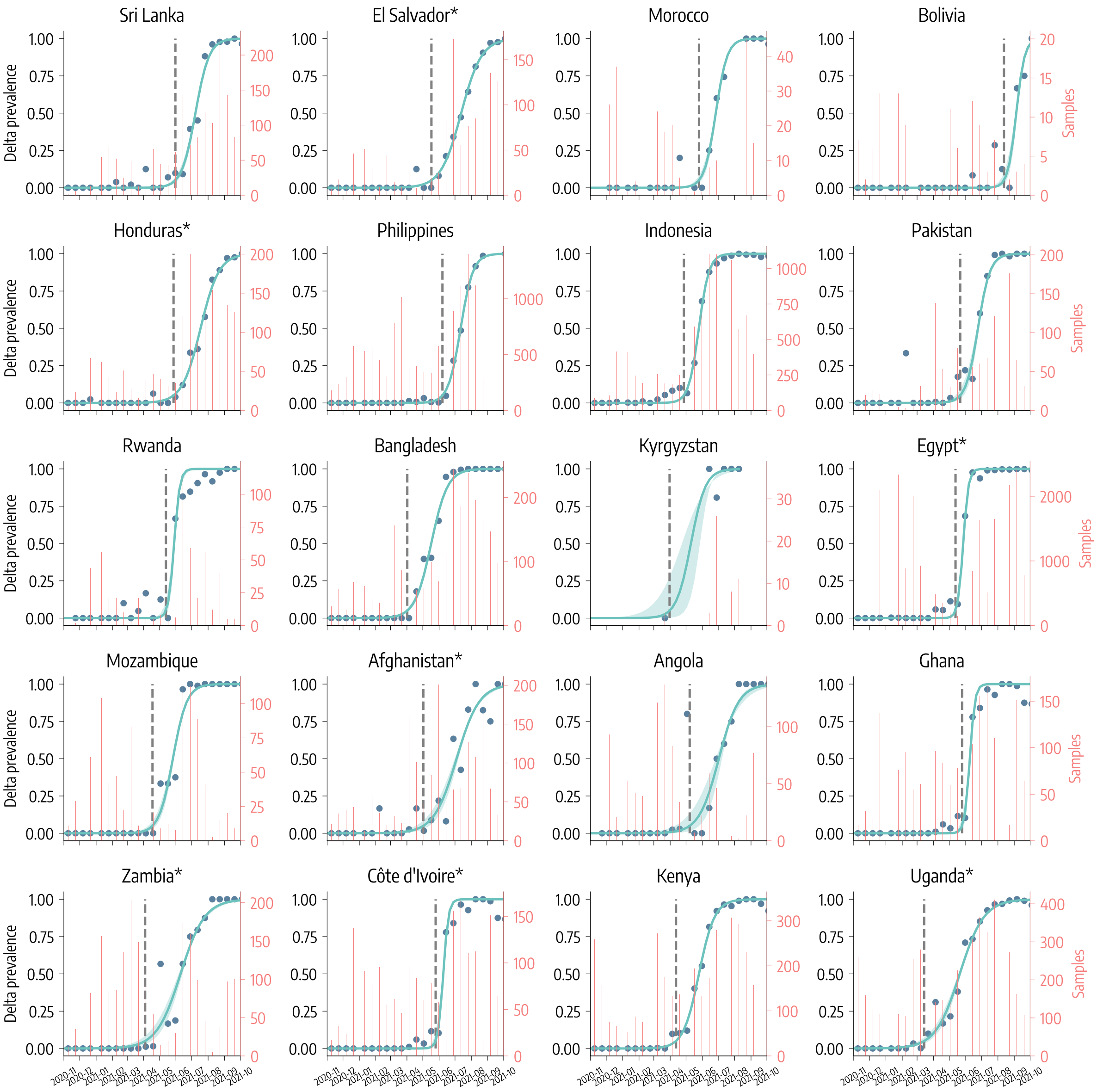

Supplementary Figure 3: **Introduction of Delta variant.** We represent the actual fraction of Delta samples and the fitted Delta prevalence (median and 90% confidence intervals). We also display the total number of samples processed per week. For countries denoted by * data are not available, therefore in those cases the fit is performed on all samples from neighbouring countries for which data are available.

with US rates lie in the middle between those obtained with Israel and EU rates. The early rollout in US was quicker than EU but slower than Israel.

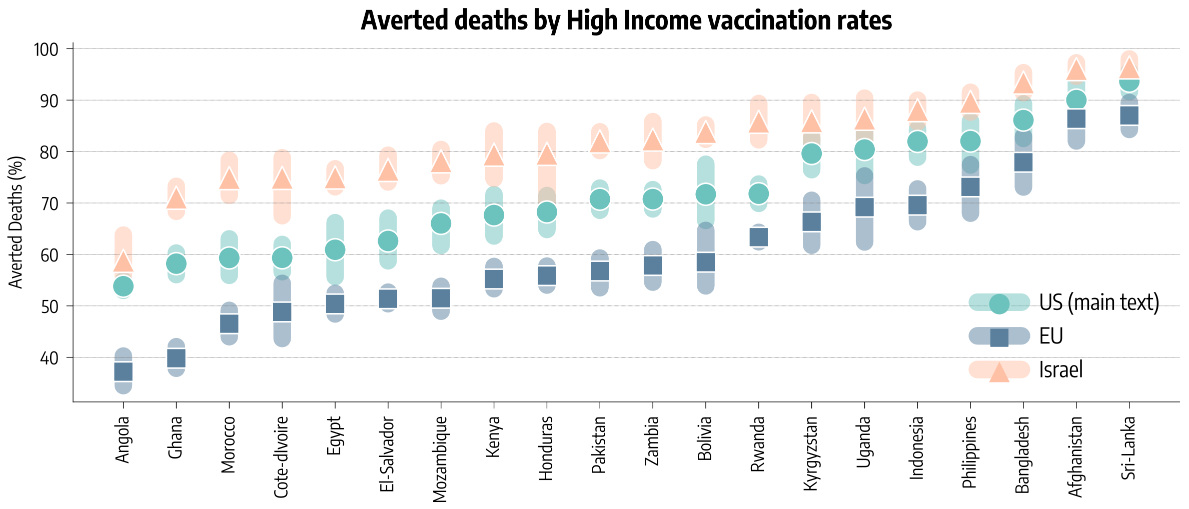

Supplementary Figure 4: **Averted Deaths with High Income regions vaccination rates.** Averted deaths (median and inter-quartile range computed over 1000 independent model realizations) expressed as a percentage with respect to the factual vaccination baseline using the vaccination rates of the United States, European Union, and Israel.

6.2 Earlier start of factual vaccinations

As a second counterfactual analysis, we anticipate the factual vaccination campaign in the LMIC in order to match the start of vaccine rollout in high income settings. As new starting date we choose the 2020/12/14, when COVID-19 vaccinations started in United States. If the shift of vaccination data causes missing data at the end of the time series of LMIC, we fill it considering the average number of doses administered during the last 7 days. It is important to stress how this counterfactual does not increases the number of doses nor the rate of vaccination. It is a solid shift of the starting date to an earlier point.

6.3 NPIs increase

We consider simulations in which we modify the factual NPIs quantified with the COVID-19 Community Mobility Report. More in detail, given the contacts reduction factor of week t $c(t)$, in the new simulations with $X\%$ additional NPIs the new factor will be $c'(t) = c(t)(1-X/100)$. The increase of NPIs is modeled as a contacts reduction factors after week 51 of 2020, a proxy date for the start of vaccinations in the United States. Indeed, our goal is to estimate the additional amount of NPIs needed to match the number of deaths averted when applying vaccination rates of a high income setting such as US. Finally, the increase of NPIs is sustained for a limited number of weeks. We explore multiple scenarios with NPIs that are increased in the range of 5% to 95% and that are sustained for a number of weeks between 4 and 40 after week 51 of 2020. We run additional simulations in which we modify the NPIs as described and we compute the fraction of averted deaths with respect to simulations with factual NPIs and vaccine rollout.

7 Estimating the impact of the factual vaccination campaigns

In Figure 1-A we have shown the evolution of the percentage of partially and fully vaccinated in the twenty LMIC countries up to 2021/10/01. As clear from the graphs, there is a high level of heterogeneity. We go from fractions of fully vaccinated above 50% in Sri Lanka and Morocco, to values below 10% in Kyrgyzstan, Mozambique, Egypt, and even below 1% in Uganda. Honduras, Bolivia, Indonesia are around the middle between these two groups with a fraction of fully vaccinated between 20% and 30%. When interpreting the numbers it is important to recognize the differences in terms of populations. Indonesia and Pakistan are the largest with 273M and 220M people respectively. El Salvador and Kyrgyzstan are the smallest with around 6M residents each. Hence, the differences in terms of the absolute number of vaccinated individuals and doses administered span several orders of magnitudes among these countries.

In Figure 5, we show the real data of confirmed deaths (dark blue dots). In most countries, the latest epidemic wave, caused by the Delta variant, was, unfortunately, the most deadly. Clearly in contrast with observations across high income countries where, despite the increased transmissibility and severity, the Delta wave was strongly limited by high vaccination rates with respect to the previous [32, 33]. This observation is a first clear hint about the impact that vaccines could have had in these settings. In the plots, we also report the median and confidence intervals of our fits (light green lines and shaded areas). Across the board, the model can capture the evolution of the pandemic with accuracy. Each plot reports also the model's prediction of what would have happened in total absence of vaccines (red dashed lines and shaded areas). In particular, we run the model keeping all the same fitted parameters, NPIs, but remove all doses administered. In doing so, we provide estimates of the impact of the factual vaccines in each country. Again, we find large heterogeneity induced by the radically different vaccination coverage. Countries that managed to vaccinate more, such as Sri Lanka, El Salvador, and Morocco show the largest differences between the real evolution of confirmed deaths and those in the hypothetical scenario without vaccines (i.e., baseline). Conversely, in countries such as Kenya, and Uganda, that have a minimal vaccination coverage, the differences are very small. Figure 6 confirms this picture but provides a more clear estimation of the impact of vaccines. We plot the deaths averted by the factual vaccination campaigns (median) versus the fraction of individuals fully vaccinated as of 2021/10/01 in the LMIC considered. Here, averted deaths are computed with respect to the baseline simulations without vaccines administered. We observe a high correlation between averted deaths and vaccination coverage (Pearson coefficient $r = 0.93$, $p_{value} < 0.001$). In Sri Lanka, Morocco and El Salvador the vaccine rollout averted more than 50% of the deaths with respect to the baseline. In Bolivia, Indonesia, Honduras, and Philippines the numbers are lower but still significant. Finally, we observe the group of countries where the doses administered are very limited ($< 10\%$) but their impact is still positive and not negligible. While interpreting the results and comparing countries it is important to stress how the model is fitted separately to each nation. Hence, some values of the free parameters such as the effective transmissibility of the strains circulating might be estimated as slightly different even though they refer to the same variants. For example, the posterior distribution for the relative transmissibility advantage of the Delta variant with respect to Alpha peaks at 1.6 in El Salvador while at 2.7 in Sri Lanka. These are effective parameters selected based on the available data. As such, they factor in many behavioral factors that are not explicitly modeled. Examples are the relations among mobility reduction, contact rates modifications, and infections. These might differ in different contexts/environments and affect the scenarios modelled here.

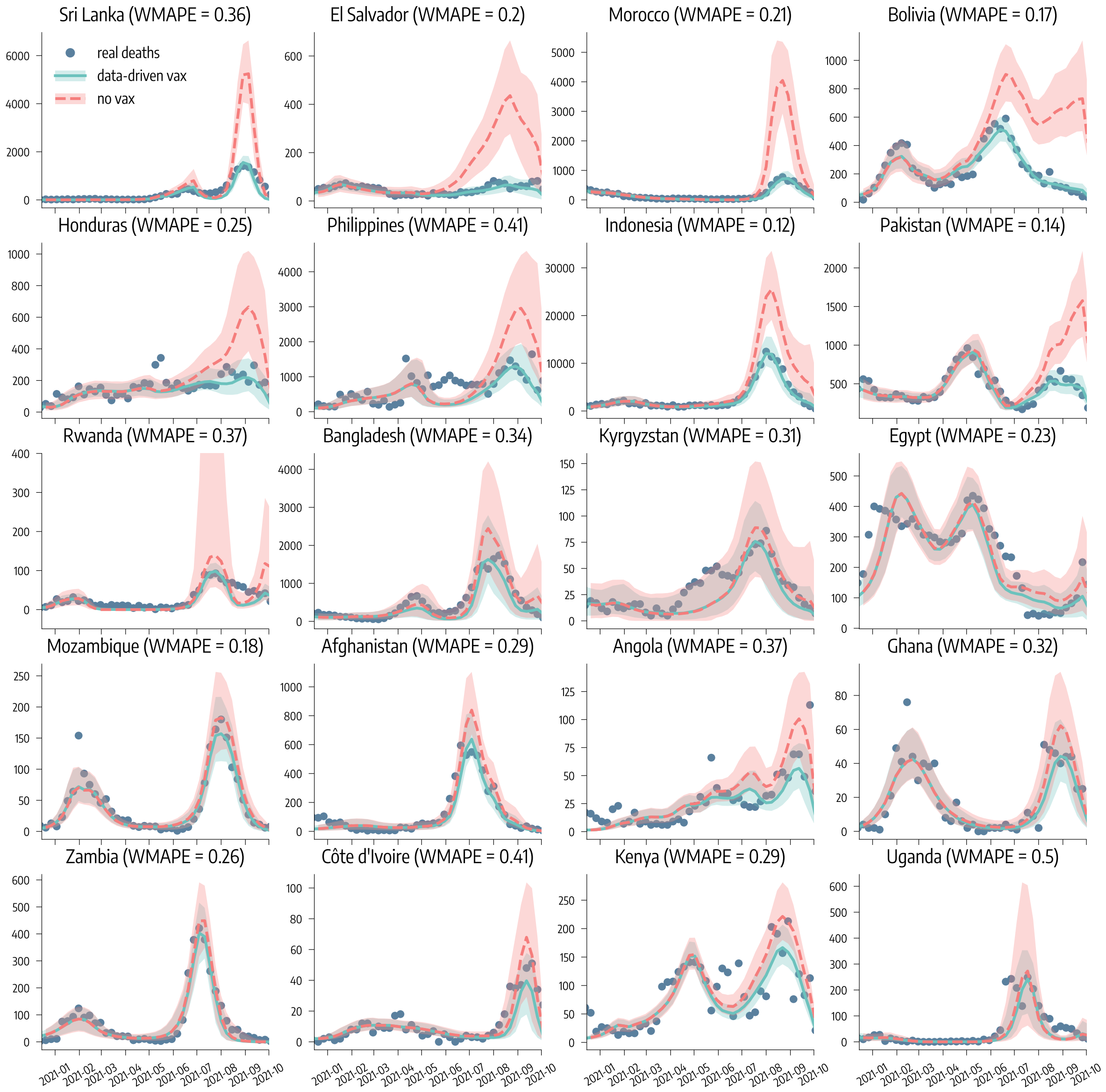

Supplementary Figure 5: **Calibration results and impact of factual vaccinations in LMIC**. We show the actual number of weekly deaths (blue dots) and the simulated weekly deaths via the calibrated model (median and 90% confidence intervals). We also show the estimated number of weekly deaths in absence of vaccines (red dashed line).

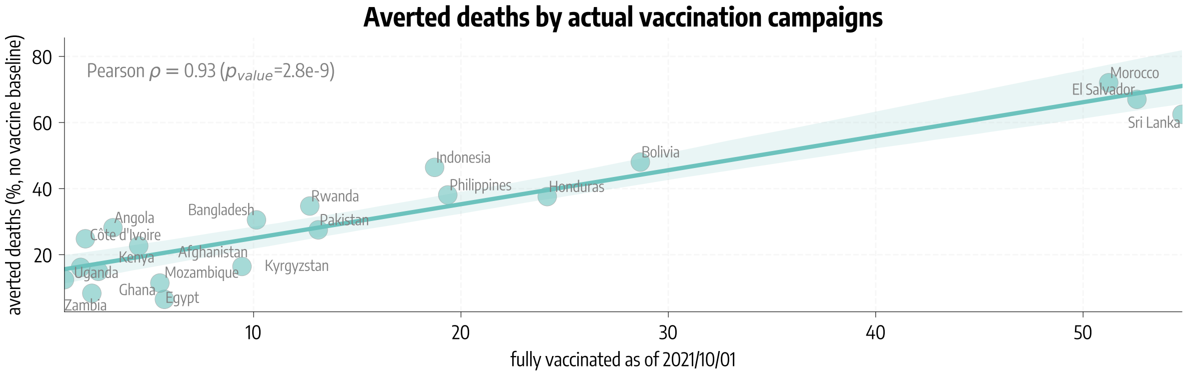

Supplementary Figure 6: **Deaths averted by factual vaccination campaigns in LMIC**. We show the estimated percentage of averted deaths (median) with respect to baseline simulations without vaccines versus the percentage of individuals fully vaccinated as of 2021/10/01 in the LMIC considered. Regression line with 95% confidence intervals is displayed. Figures also report the results (coefficient and p-value) of the two-sided Pearson correlation test.

8 Model with time-varying reporting of deaths

We extend the model setup presented above to account for time-varying ability of countries to detect deaths. Indeed, despite the period considered is relatively short and does not include the first uncertain months of the Pandemic, we acknowledge possible changes in deaths reporting rates. Indeed, countries may have improved in time their COVID-19 surveillance, or, on the other hand, reporting may have degraded during the chaos brought by the Delta wave. To assess how this may affect our findings, here we present a model that, instead of a single one, features two deaths reporting parameter: one for the first and one for the second half of the period. In principle, one may add more than two reporting parameters related to smaller fraction of the simulation, but that would significantly increase model's complexity. We repeat the calibration step for each country using as a prior distribution of the first deaths reporting parameter the 95% confidence interval of the posterior distribution of the single reporting parameter of the model presented in the main text. The second reporting parameter can instead vary in a range of +/- 20 percentage points with respect to the first one (this percentage deviation is a new parameter that is calibrated in the ABC-SMC process). As we can see from Tab. 6, where we show the posterior distribution (median and IQR) for the two parameters, the model captures, in general, an improvement in the ability of countries to detect deaths. Nonetheless, this has little impact on our main findings. Tab. 7 and Tab. 8 show, the estimated averted deaths in the scenario with US-equivalent vaccine availability and with US-equivalent vaccine rollout start date respectively. We see that, the estimates from the 2-factors model always fall in the IQR range estimated with the single factor model, with the exceptions of Philippines, Bangladesh, and Ghana. Nonetheless, in these cases the new estimates fall into the 90% confidence intervals. We conclude that this change in modeling setup do not significantly impact the findings presented in the main text.

**Country**	**Deaths Reported (%) -** 1st	**Deaths Reported (%) -** 2nd
Sri Lanka	33.54 [27.36, 26.35]	33.11 [26.35, 40.32]
El Salvador	50.23 [41.42, 43.13]	51.99 [43.13, 62.84]
Morocco	37.3 [30.27, 33.85]	42.05 [33.85, 50.17]
Bolivia	89.86 [84.84, 83.81]	91.14 [83.81, 99.0]
Honduras	63.47 [53.64, 58.49]	67.42 [58.49, 77.61]
Philippines	33.24 [29.49, 42.8]	46.98 [42.8, 51.7]
Indonesia	67.58 [57.42, 58.96]	70.53 [58.96, 82.59]
Pakistan	53.8 [43.22, 47.88]	58.29 [47.88, 71.51]
Rwanda	46.44 [35.61, 40.42]	51.35 [40.42, 64.21]
Bangladesh	14.4 [11.12, 18.46]	23.16 [18.46, 28.15]
Kyrgyzstan	47.5 [35.01, 41.9]	53.66 [41.9, 66.96]
Egypt	34.68 [31.93, 35.3]	39.44 [35.3, 43.6]
Mozambique	7.08 [5.85, 4.15]	5.22 [4.15, 6.45]
Afghanistan	24.85 [19.41, 20.41]	26.52 [20.41, 32.62]
Angola	3.96 [3.22, 3.16]	3.93 [3.16, 4.76]
Ghana	1.62 [1.48, 1.0]	1.0 [1.0, 1.0]
Zambia	22.44 [17.81, 16.53]	21.06 [16.53, 26.46]
Côte d'Ivoire	1.59 [1.3, 1.62]	2.2 [1.62, 2.82]
Kenya	7.85 [6.32, 7.84]	9.5 [7.84, 11.16]
Uganda	13.72 [10.32, 17.81]	23.65 [18.82, 28.19]

Supplementary Table 6: Posterior distributions (median and IQR) of percentage of deaths reported in the 1st and 2nd half of the simulations obtained with the two deaths-reporting parameters model.

	Averted Deaths (Raw Number)		Averted Deaths (%)	
Country	*1-Factor Model*	*2-Factors Model*	*1-Factor Model*	*2-Factors Model*
Sri Lanka	19.1K [15.2K, 25.0K]	18.4K [14.9K, 22.4K]	62.61 [58.77, 67.05]	62.8 [59.2, 66.46]
El Salvador	2.2K [1.7K, 2.7K]	2.2K [1.8K, 2.6K]	59.31 [55.94, 63.02]	59.37 [56.3, 62.31]
Morocco	12.1K [9.7K, 15.5K]	10.7K [8.8K, 12.9K]	67.65 [63.59, 71.62]	65.66 [60.8, 70.56]
Bolivia	7.8K [7.2K, 8.3K]	7.6K [7.1K, 8.1K]	70.76 [68.86, 72.59]	70.37 [68.26, 72.39]
Honduras	5.8K [4.8K, 7.1K]	5.4K [4.7K, 6.3K]	60.91 [55.68, 66.17]	61.13 [56.23, 66.61]
Philippines	51.7K [43.2K, 61.3K]	42.5K [38.0K, 47.6K]	79.61 [76.48, 82.61]	75.11 [73.68, 76.76]
Indonesia	149.3K [122.5K, 182.7K]	137.5K [118.7K, 163.0K]	81.97 [78.91, 84.36]	81.51 [78.86, 83.71]
Pakistan	28.3K [21.1K, 38.2K]	22.6K [18.3K, 27.9K]	71.8 [69.93, 73.68]	70.79 [68.8, 72.48]
Rwanda	1.7K [1.1K, 2.6K]	1.4K [1.0K, 1.9K]	82.03 [77.39, 85.88]	80.36 [76.18, 84.08]
Bangladesh	93.2K [71.0K, 122.9K]	66.6K [54.5K, 82.7K]	86.12 [82.55, 89.27]	83.68 [80.34, 86.81]
Kyrgyzstan	1.7K [1.2K, 2.5K]	1.6K [1.2K, 2.1K]	80.39 [75.36, 85.0]	78.28 [73.42, 82.56]
Egypt	16.6K [14.5K, 18.8K]	14.5K [13.1K, 16.2K]	58.21 [56.08, 60.27]	55.98 [54.28, 57.95]
Mozambique	20.6K [15.4K, 26.8K]	21.3K [18.0K, 27.1K]	68.25 [64.86, 71.45]	70.05 [67.3, 72.72]
Afghanistan	18.5K [14.3K, 24.1K]	16.2K [13.4K, 20.3K]	93.67 [91.64, 95.54]	93.39 [91.22, 95.32]
Angola	15.4K [12.0K, 20.9K]	12.4K [10.3K, 15.8K]	53.82 [53.02, 54.9]	55.2 [54.71, 55.75]
Ghana	20.0K [15.6K, 26.0K]	35.7K [33.1K, 38.6K]	59.34 [56.64, 61.93]	63.36 [61.36, 65.24]
Zambia	12.9K [10.0K, 15.5K]	11.1K [8.8K, 13.8K]	71.69 [66.61, 77.53]	73.94 [69.8, 77.9]
Côte d'Ivoire	15.3K [12.5K, 18.0K]	13.5K [11.3K, 18.6K]	66.03 [61.75, 68.99]	66.81 [63.64, 69.17]
Kenya	30.8K [23.4K, 41.1K]	24.4K [21.0K, 28.7K]	70.74 [68.59, 72.89]	71.1 [69.39, 73.38]
Uganda	4.5K [3.3K, 5.9K]	4.9K [3.4K, 7.0K]	90.01 [86.85, 93.58]	87.35 [82.3, 91.24]

Supplementary Table 7: Averted deaths - expressed both as raw number and as percentage - estimated by the model with single deaths reporting parameter (presented in the main text) and by the model with two parameters in a US-equivalent vaccine availability scenario.

	**Averted Deaths (Raw Number)**		**Averted Deaths (%)**	
Country	*1-Factor Model*	*2-Factors Model*	*1-Factor Model*	*2-Factors Model*
Sri Lanka	13.5K [10.7K, 17.7K]	13.0K [10.5K, 16.0K]	44.29 [42.44, 46.39]	44.4 [42.96, 46.11]
El Salvador	1.7K [1.4K, 2.2K]	1.7K [1.4K, 2.0K]	46.86 [43.62, 50.54]	46.78 [44.22, 49.49]
Morocco	9.0K [7.1K, 11.6K]	7.7K [6.4K, 9.4K]	50.25 [45.17, 55.55]	48.05 [42.77, 53.7]
Bolivia	3.8K [3.5K, 4.0K]	3.7K [3.4K, 3.9K]	34.14 [32.81, 35.48]	33.88 [32.55, 35.19]
Honduras	3.1K [2.5K, 3.7K]	2.8K [2.5K, 3.2K]	31.94 [30.37, 33.57]	31.72 [30.04, 33.5]
Philippines	29.4K [24.9K, 34.1K]	24.0K [21.5K, 26.8K]	45.02 [41.61, 48.56]	42.5 [40.8, 44.24]
Indonesia	38.0K [31.5K, 45.7K]	35.3K [30.6K, 41.4K]	20.61 [19.69, 21.73]	20.67 [19.88, 21.54]
Pakistan	9.2K [6.8K, 12.1K]	7.3K [5.9K, 8.9K]	23.08 [21.63, 24.51]	22.71 [21.24, 24.07]
Rwanda	0.9K [0.5K, 1.4K]	0.7K [0.5K, 1.1K]	42.45 [33.25, 51.13]	42.13 [32.78, 51.04]
Bangladesh	22.0K [15.3K, 31.3K]	14.5K [11.5K, 18.5K]	20.87 [16.79, 23.74]	18.15 [15.8, 20.68]
Kyrgyzstan	0.8K [0.6K, 1.2K]	0.8K [0.6K, 1.0K]	37.83 [33.75, 42.75]	36.71 [32.87, 40.98]
Egypt	1.8K [1.5K, 2.2K]	1.5K [1.2K, 1.9K]	6.25 [5.19, 7.24]	5.84 [4.83, 6.81]
Mozambique	8.5K [6.4K, 11.0K]	9.0K [7.5K, 11.5K]	28.26 [27.08, 29.51]	29.23 [28.3, 30.14]
Afghanistan	2.9K [2.3K, 3.9K]	2.5K [2.0K, 3.2K]	14.73 [13.64, 16.05]	14.34 [13.2, 15.53]
Angola	6.4K [5.0K, 8.6K]	4.9K [4.0K, 6.2K]	22.38 [21.84, 22.83]	21.63 [21.1, 22.15]
Ghana	4.9K [3.8K, 6.3K]	8.0K [7.2K, 8.8K]	14.41 [13.27, 15.59]	14.32 [13.34, 15.05]
Zambia	4.0K [3.1K, 4.8K]	3.3K [2.7K, 4.2K]	22.08 [20.86, 23.49]	22.38 [21.22, 23.47]
Côte d'Ivoire	3.8K [3.1K, 4.4K]	3.4K [2.8K, 4.6K]	16.29 [15.52, 17.05]	16.51 [15.85, 17.21]
Kenya	8.4K [6.4K, 11.3K]	6.6K [5.6K, 7.9K]	19.36 [18.87, 19.84]	19.23 [18.74, 19.74]
Uganda	0.9K [0.5K, 1.2K]	0.9K [-0.4K, 2.3K]	18.51 [12.62, 23.98]	16.93 [-8.57, 35.21]

Supplementary Table 8: Averted deaths - expressed both as raw number and as percentage - estimated by the model with single deaths reporting parameter (presented in the main text) and by the model with two parameters in a scenario with US-equivalent start of vaccine rollout.

9 Countries demographic

To better characterize the 20 countries selected, in Fig. 7 we show the share of population in different age brackets (from $0-10$ to $80+$). The figure also shows the estimated average age for all countries. The average age in the select countries is 26, with some dispersion around this value. Uganda is the youngest country with an average age of 20.6, while the oldest among those considered is Sri Lanka, with an average age of 34.8. In Fig. 8 we show for each country the overall contact matrices that we use to inform the epidemic model taken from Ref. [8]. Qualitatively, we do not notice dramatic differences in terms of contact rates. Indeed, among the countries considered contact intensities is significantly higher in younger age groups. These small differences are also confirmed by Fig. 9 where we show the spectral radius (i.e., absolute value of the largest eigenvalue) of the normalized contact matrix, whose element c'_{ij} is defined as $c'_{ij} = \frac{N_i}{N_j} c_{ij}$, where N_i (N_j) is the number of individuals i age group i (j) and c_{ij} is the i, j element of the original contact matrix. The basic reproductive number R_0 of a SEIR-like model is proportional to the spectral radius of such matrices. Hence, this quantity act as the effective strength of contacts relevant for epidemic spreading. In the countries considered, the spectral radius is on average 13.41, spanning from a maximum of 14.07 in Sri Lanka, to a minimum 12.95 in Angola (marking a maximum-minimum difference of only 8.3% respect to the average).

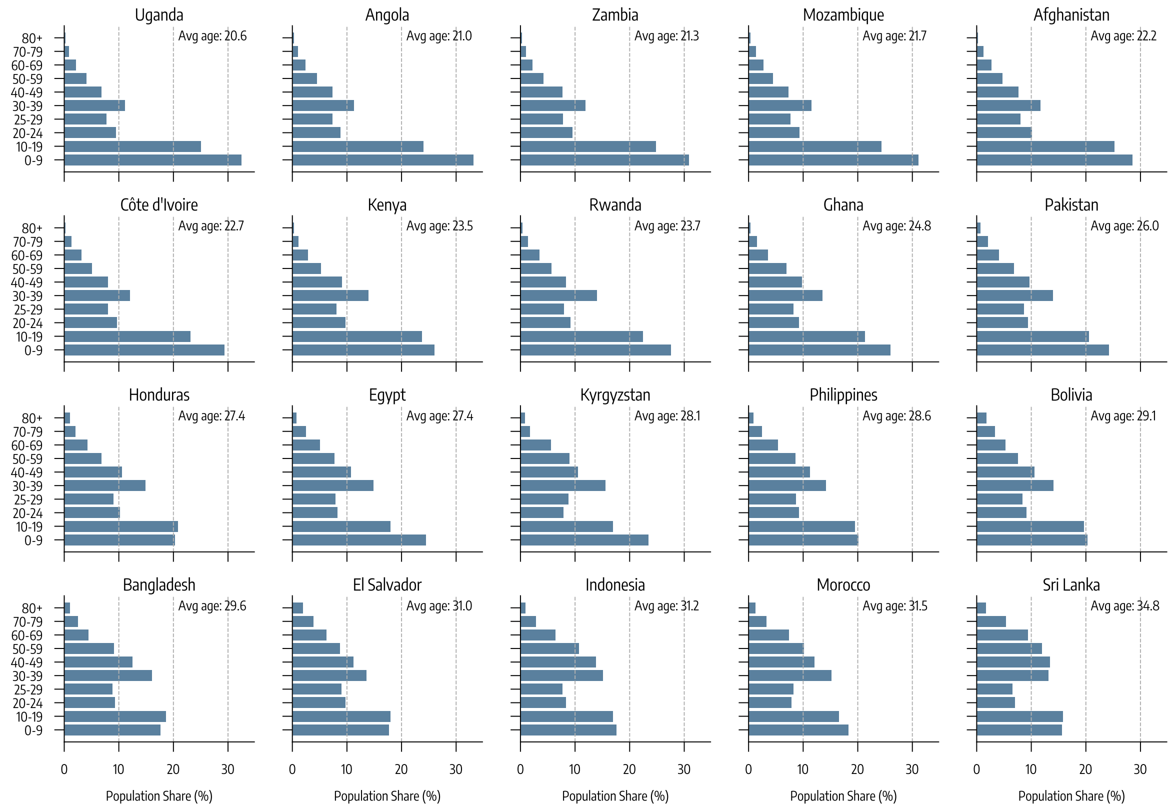

Supplementary Figure 7: **Age group distribution.** We show for the 20 selected countries the population share in each age group, from $0-10$ to $80+$. In the figure we also report the average age of each country. Country are ordered from youngest to oldest one.

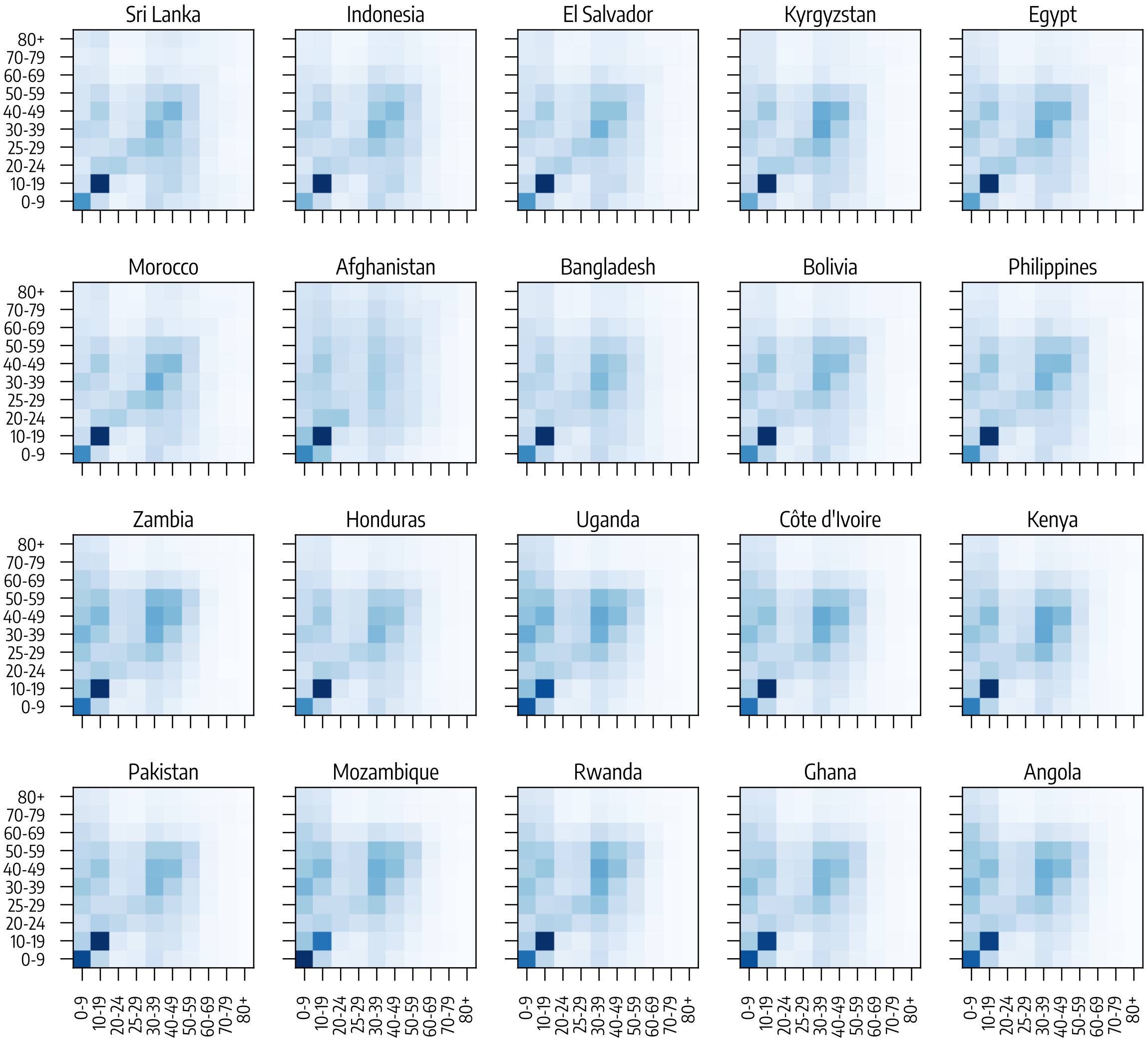

Supplementary Figure 8: **Contact Matrices** We show the overall contact matrix for each country.

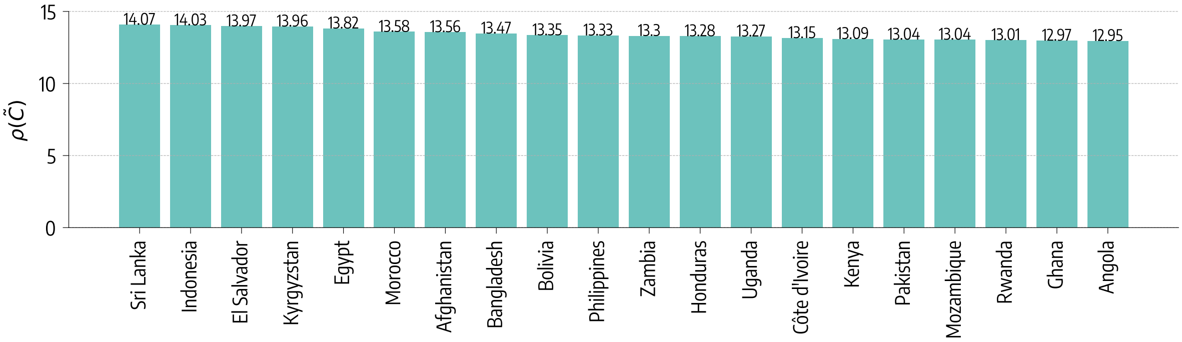

Supplementary Figure 9: **Spectral radius** We show the spectral radius (i.e., largest eigenvalue) of the normalized contact matrix of each country.

Supplementary References

[1] United Nations, Department of Economic and Social Affairs, Population Division. World Population Prospects: The 2019 Revision. https://population.un.org/wpp/Download/Metadata/Documentation/, 2020. Accessed: 2020-11-30.

[2] COVID-19 Data Repository by the Center for Systems Science and Engineering (CSSE) at Johns Hopkins University. https://github.com/CSSEGISandData/COVID-19, 2021.

[3] Global Dashbord for Vaccine Equity. https://data.undp.org/vaccine-equity/, 2021. Accessed: 2021-11-30.

[4] Coronavirus (COVID-19) Vaccinations. https://ourworldindata.org/covid-vaccinations, 2020. Accessed: 2020-11-30.

[5] Jantien A Backer, Don Klinkenberg, and Jacco Wallinga. Incubation period of 2019 novel coronavirus (2019-nCoV) infections among travellers from Wuhan, China, 20–28 January 2020. *Eurosurveillance*, 25(5), 2020.

[6] Stephen M. Kissler, Christine Tedijanto, Edward Goldstein, Yonatan H. Grad, and Marc Lipsitch. Projecting the transmission dynamics of SARS-CoV-2 through the postpandemic period. *Science*, 368(6493):860–868, 2020.

[7] Robert Verity, Lucy Okell, Ilaria Dorigatti, Peter Winskill, Charles Whittaker, Natsuko Imai, Gina Cuomo-Dannenburg, Hayley Thompson, Patrick Walker, Han Fu, Amy Dighe, Jamie Griffin, Marc Baguelin, Sangeeta Bhatia, Adhiratha Boonyasiri, Anne Cori, Zulma M. Cucunubá, Rich FitzJohn, Katy Gaythorpe, and Neil Ferguson. Estimates of the severity of coronavirus disease 2019: a model-based analysis. *The Lancet Infectious Diseases*, 20, 03 2020.

[8] Dina Mistry, Maria Litvinova, Ana Pastore y Piontti, Matteo Chinazzi, Laura Fumanelli, Marcelo F C Gomes, Syed A Haque, Quan-Hui Liu, Kunpeng Mu, Xinyue Xiong, M Elizabeth Halloran, Ira M Longini, Stefano Merler, Marco Ajelli, and Alessandro Vespignani. Inferring high-resolution human mixing patterns for disease modeling. *Nature Communications*, 12(1):323, 2021.

[9] Duygu Balcan, Bruno Gonçalves, Hao Hu, José J. Ramasco, Vittoria Colizza, and Alessandro Vespignani. Modeling the spatial spread of infectious diseases: The GLobal Epidemic and Mobility computational model. *Journal of Computational Science*, 1(3):132 – 145, 2010.

[10] Ben S Cooper, Richard J Pitman, W John Edmunds, and Nigel J Gay. Delaying the international spread of pandemic influenza. *PLoS Med*, 3(6):e212, 2006.

[11] Julia Shapiro, Natalie E. Dean, Zachary J. Madewell, Yang Yang, M.Elizabeth Halloran, and Ira Longini. Efficacy Estimates for Various COVID-19 Vaccines: What we Know from the Literature and Reports. *medRxiv*, 2021.

[12] Kate M Bubar, Kyle Reinholt, Stephen M Kissler, Marc Lipsitch, Sarah Cobey, Yonatan H Grad, and Daniel B Larremore. Model-informed COVID-19 vaccine prioritization strategies by age and serostatus. *Science*, 371(6532):916–921, 2021.

[13] Laura Matrajt, Julia Eaton, Tiffany Leung, and Elizabeth R. Brown. Vaccine optimization for COVID-19: Who to vaccinate first? *Science Advances*, 7(6), 2020.

[14] Wei Wang, Qianhui Wu, Juan Yang, Kaige Dong, Xinghui Chen, Xufang Bai, Xinhua Chen, Zhiyuan Chen, Cécile Viboud, Marco Ajelli, et al. Global, regional, and national estimates of target population sizes for COVID-19 vaccination: descriptive study. *bmj*, 371, 2020.

[15] Edouard Mathieu, Hannah Ritchie, Esteban Ortiz-Ospina, Max Roser, Joe Hasell, Cameron Appel, Charlie Giattino, and Lucas Rodés-Guirao. A global database of COVID-19 vaccinations. *Nature Human Behaviour*, 5(7):947–953, 2021.

[16] CoVariants. https://covariants.org, 2021.

[17] Nextstrain. https://nextstrain.org, 2021.

[18] Stefan Elbe and Gemma Buckland-Merrett. Data, disease and diplomacy: GISAID's innovative contribution to global health. *Global Challenges*, 1(1):33–46, 2017.

[19] Baisheng Li, Aiping Deng, Kuibiao Li, Yao Hu, Zhencui Li, Yaling Shi, Qianling Xiong, Zhe Liu, Qianfang Guo, Lirong Zou, et al. Viral infection and transmission in a large, well-traced outbreak caused by the SARS-CoV-2 Delta variant. *Nature communications*, 13(1):460, 2022.

[20] Katherine A Twohig, Tommy Nyberg, Asad Zaidi, Simon Thelwall, Mary A Sinnathamby, Shirin Aliabadi, Shaun R Seaman, Ross J Harris, Russell Hope, Jamie Lopez-Bernal, et al. Hospital admission and emergency care attendance risk for SARS-CoV-2 delta (B. 1.617. 2) compared with alpha (B. 1.1. 7) variants of concern: a cohort study. *The Lancet Infectious Diseases*, 22(1):35–42, 2022.

[21] Amanda Minter and Renata Retkute. Approximate Bayesian Computation for infectious disease modelling. *Epidemics*, 29:100368, 2019.

[22] Mikael Sunnåker, Alberto Giovanni Busetto, Elina Numminen, Jukka Corander, Matthieu Foll, and Christophe Dessimoz. Approximate Bayesian Computation. *PLOS Computational Biology*, 9(1):1–10, 01 2013.

[23] Centers for Disease Control and Prevention, COVID-19 Pandemic Planning Scenarios. `https://www.cdc.gov/coronavirus/2019-ncov/hcp/planning-scenarios.html#table-1`, 2021. Accessed 2021/02/02.

[24] Emmanuel Klinger, Dennis Rickert, and Jan Hasenauer. pyABC: distributed, likelihood-free inference. *Bioinformatics*, 34(20):3591–3593, 05 2018.

[25] Google LLC "Google COVID-19 Community Mobility Reports". `https://www.google.com/covid19/mobility/`, 2020. Accessed: 2021-08-01.

[26] Nuria Oliver, Bruno Lepri, Harald Sterly, Renaud Lambiotte, Sébastien Deletaille, Marco De Nadai, Emmanuel Letouzé, Albert Ali Salah, Richard Benjamins, Ciro Cattuto, Vittoria Colizza, Nicolas de Cordes, Samuel P. Fraiberger, Till Koebe, Sune Lehmann, Juan Murillo, Alex Pentland, Phuong N Pham, Frédéric Pivetta, Jari Saramäki, Samuel V. Scarpino, Michele Tizzoni, Stefaan Verhulst, and Patrick Vinck. Mobile phone data for informing public health actions across the covid-19 pandemic life cycle. *Science Advances*, 6(23), 2020.

[27] Laura Alessandretti. What human mobility data tell us about COVID-19 spread. *Nature Reviews Physics*, 4(1):12–13, 2022.

[28] Serina Chang, Emma Pierson, Pang Wei Koh, Jaline Gerardin, Beth Redbird, David Grusky, and Jure Leskovec. Mobility network models of COVID-19 explain inequities and inform reopening. *Nature*, 589(7840):82–87, 2021.

[29] Moritz U. G. Kraemer, Chia-Hung Yang, Bernardo Gutierrez, Chieh-Hsi Wu, Brennan Klein, David M. Pigott, Louis du Plessis, Nuno R. Faria, Ruoran Li, William P. Hanage, John S. Brownstein, Maylis Layan, Alessandro Vespignani, Huaiyu Tian, Christopher Dye, Oliver G. Pybus, and Samuel V. Scarpino and. The effect of human mobility and control measures on the COVID-19 epidemic in China. *Science*, 368(6490):493–497, mar 2020.

[30] Nicolò Gozzi, Michele Tizzoni, Matteo Chinazzi, Leo Ferres, Alessandro Vespignani, and Nicola Perra. Estimating the effect of social inequalities on the mitigation of COVID-19 across communities in Santiago de Chile. *Nature Communications*, 12(1):2429, 2021.

[31] Pauli Virtanen, Ralf Gommers, Travis E. Oliphant, Matt Haberland, Tyler Reddy, David Cournapeau, Evgeni Burovski, Pearu Peterson, Warren Weckesser, Jonathan Bright, Stéfan J. van der Walt, Matthew Brett, Joshua Wilson, K. Jarrod Millman, Nikolay Mayorov, Andrew R. J. Nelson, Eric Jones, Robert Kern, Eric Larson, C J Carey, İlhan Polat, Yu Feng, Eric W. Moore, Jake VanderPlas, Denis Laxalde, Josef Perktold, Robert Cimrman, Ian Henriksen, E. A. Quintero, Charles R. Harris, Anne M. Archibald, Antônio H. Ribeiro, Fabian Pedregosa, Paul van Mulbregt, and SciPy 1.0 Contributors. SciPy 1.0: Fundamental Algorithms for Scientific Computing in Python. *Nature Methods*, 17:261–272, 2020.

[32] Nicolò Gozzi, Matteo Chinazzi, Jessica T Davis, Kunpeng Mu, Ana Pastore y Piontti, Marco Ajelli, Nicola Perra, and Alessandro Vespignani. Anatomy of the first six months of COVID-19 Vaccination Campaign in Italy. *PLoS Computational Biology*, 18(5):e1010146, 2022.

[33] Valentina Marziano, Giorgio Guzzetta, Alessia Mammone, Flavia Riccardo, Piero Poletti, Filippo Trentini, Mattia Manica, Andrea Siddu, Antonino Bella, Paola Stefanelli, et al. The effect of COVID-19 vaccination in Italy and perspectives for living with the virus. *Nature communications*, 12(1):1–8, 2021.
