# Supplementary material for: Estimating the impact of COVID-19 vaccine inequities: a modeling study
Source: Nat Commun. 2023 Jun 6;14:3272. doi: 10.1038/s41467-023-39098-w (PMC10241610; doi:10.1038/s41467-023-39098-w)
Supplement: Supplementary file 3 — Reporting Summary [file 41467_2023_39098_MOESM3_ESM.pdf]

nature portfolio

Corresponding author(s): Nicola Perra

Last updated by author(s): May 16, 2023

Reporting Summary

Nature Portfolio wishes to improve the reproducibility of the work that we publish. This form provides structure for consistency and transparency in reporting. For further information on Nature Portfolio policies, see our Editorial Policies and the Editorial Policy Checklist.

Statistics

For all statistical analyses, confirm that the following items are present in the figure legend, table legend, main text, or Methods section.

n/a	Confirmed	
☐	☒	The exact sample size (*n*) for each experimental group/condition, given as a discrete number and unit of measurement
☒	☐	A statement on whether measurements were taken from distinct samples or whether the same sample was measured repeatedly
☐	☒	The statistical test(s) used AND whether they are one- or two-sided<br>*Only common tests should be described solely by name; describe more complex techniques in the Methods section.*
☐	☒	A description of all covariates tested
☐	☒	A description of any assumptions or corrections, such as tests of normality and adjustment for multiple comparisons
☐	☒	A full description of the statistical parameters including central tendency (e.g. means) or other basic estimates (e.g. regression coefficient) AND variation (e.g. standard deviation) or associated estimates of uncertainty (e.g. confidence intervals)
☐	☒	For null hypothesis testing, the test statistic (e.g. *F*, *t*, *r*) with confidence intervals, effect sizes, degrees of freedom and *P* value noted<br>*Give P values as exact values whenever suitable.*
☐	☒	For Bayesian analysis, information on the choice of priors and Markov chain Monte Carlo settings
☒	☐	For hierarchical and complex designs, identification of the appropriate level for tests and full reporting of outcomes
☐	☒	Estimates of effect sizes (e.g. Cohen's *d*, Pearson's *r*), indicating how they were calculated

Our web collection on statistics for biologists contains articles on many of the points above.

Software and code

Policy information about availability of computer code

Data collection	The codes are written in Python 3.8.8
Data analysis	All the analysis were performed in Python 3.8.8 using the following packages scipy 1.7.1, pyabc 0.12.9. Codes are available at the following link: https://github.com/ngozzi/vaccine-lmic

For manuscripts utilizing custom algorithms or software that are central to the research but not yet described in published literature, software must be made available to editors and reviewers. We strongly encourage code deposition in a community repository (e.g. GitHub). See the Nature Portfolio guidelines for submitting code & software for further information.

Data

Policy information about availability of data

All manuscripts must include a data availability statement. This statement should provide the following information, where applicable:

- Accession codes, unique identifiers, or web links for publicly available datasets
- A description of any restrictions on data availability
- For clinical datasets or third party data, please ensure that the statement adheres to our policy

The data used for this study is publicly available. Data on vaccinations can be downloaded at https://ourworldindata.org/covid-vaccinations, demographic data at

https://population.un.org/wpp/Download/Metadata/Documentation/, mobility data at https://www.google.com/covid19/mobility/, and epidemiological data at https://github.com/CSSEGISandData/COVID-19

Research involving human participants, their data, or biological material

Policy information about studies with human participants or human data. See also policy information about sex, gender (identity/presentation), and sexual orientation and race, ethnicity and racism.

Reporting on sex and gender	We do not report on sex or gender as the data used is aggregated
Reporting on race, ethnicity, or other socially relevant groupings	We do not report on race, ethnicity, or other socially relevant groupings as the data used is aggregated
Population characteristics	See below
Recruitment	We did not recruit participants but only used is publicly available and aggregated secondary data
Ethics oversight	None

Note that full information on the approval of the study protocol must also be provided in the manuscript.

Field-specific reporting

Please select the one below that is the best fit for your research. If you are not sure, read the appropriate sections before making your selection.

☐ Life sciences ☒ Behavioural & social sciences ☐ Ecological, evolutionary & environmental sciences

For a reference copy of the document with all sections, see nature.com/documents/nr-reporting-summary-flat.pdf

Behavioural & social sciences study design

All studies must disclose on these points even when the disclosure is negative.

Study description	We investigated the impact of vaccine inequities in 20 low and lower middle income countries with a data-driven, age-structured, stochastic epidemic model. The study is based on quantitative methods.
Research sample	The target population are the residents in the 20 countries investigated. We used several datasets, in particular:<br><br>Data on global vaccine inequities come from the United Nations Development Programme via their Global Futures Platform. The data detailing the timeline of vaccinations used in the simulation come from Our World in Data. The dataset provides the cumulative share of people partially and fully vaccinated against SARS-CoV-2 as function of time<br><br>Data about demographics come from the United Nation World Population Prospects. Epidemiological data are extracted from the COVID-19 Data Repository by the Center for Systems Science and Engineering (CSSE) at Johns Hopkins University and from official sources.<br><br>Data to estimate the impact of NPIs on transmission dynamics come from the COVID-19 Community Mobility Report by Google. The dataset provides the percentage change in mobility r(t) on day t. We compute r(t) by using the average of the fields "workplaces percent change from baseline", "retail and recreation percent change from baseline", and "transit stations percent change from baseline".<br><br>The data utilized in this study were employed to characterize the sociodemographic context of the countries under consideration, as well as their epidemiological conditions (including COVID-19 prevalence, immunity from prior infections, and vaccine availability). This information was then integrated into the proposed epidemic model to enhance its accuracy and realism.<br><br>Data on vaccine availability, epidemiological conditions, and demographic are related to the whole population. When it comes to the mobility data, it is not feasible to assess the representativeness and characteristics of the underlying population due to the unavailability of this information which is not shared by Google with researchers.
Sampling strategy	We have sampled 20 countries from lower and low income groups to cover all WHO regions. We used convenience sampling since our sampling strategy was guided by data availability on vaccine distribution, COVID-19 cases and deaths, SARS-CoV-2 variants prevalence, and mobility.
Data collection	We made use of secondary data only. Data used are all open access and have been downloaded from their public sources mentioned above
Timing	Our proposed epidemic model covers the period 2020/10/01 - 2021/10/01
Data exclusions	None

Non-participation	We used only secondary aggregated data, therefore this study did not require the direct involvement of participants.
Randomization	We used only secondary aggregated data, therefore the data does not allow randomization strategies at the individual level.

Reporting for specific materials, systems and methods

We require information from authors about some types of materials, experimental systems and methods used in many studies. Here, indicate whether each material, system or method listed is relevant to your study. If you are not sure if a list item applies to your research, read the appropriate section before selecting a response.

Materials & experimental systems

n/a	Involved in the study	
☒	☐	Antibodies
☒	☐	Eukaryotic cell lines
☒	☐	Palaeontology and archaeology
☒	☐	Animals and other organisms
☒	☐	Clinical data
☒	☐	Dual use research of concern
☒	☐	Plants

Methods

n/a	Involved in the study	
☒	☐	ChIP-seq
☒	☐	Flow cytometry
☒	☐	MRI-based neuroimaging
